# Crimean-Congo Hemorrhagic Fever Virus Seropositivity among Dromedary Camels, Algeria, 2020–2021

**DOI:** 10.3201/eid2912.230587

**Published:** 2023-12

**Authors:** Khaled Azzedine Guidoum, Laura Carrera-Faja, Johan Espunyes, Lola Pailler-García, Bouabdellah Benallou, Sarra Bouabdelli, Mustapha Adnane Smadi, Lounis Semara, Oscar Cabezón, Sebastián Napp

**Affiliations:** Ibn Khaldoun University, Zaârour, Algeria (K.A. Guidoum, B. Benallou, S. Bouabdelli);; Universitat Autònoma de Barcelona, Bellaterra, Spain (L. Carrera-Faja, J. Espunyes, O. Cabezón);; Institut de Recerca i Tecnologia Agroalimentàries, Bellaterra (L. Pailler-García, S. Napp);; Biotechnology Research Center, Constantine, Algeria (M.A. Smadi);; Mohamed El Bachir El Ibrahimi University, El Anceur, Algeria (L. Semara)

**Keywords:** Crimean-Congo hemorrhagic fever virus, CCHV, dromedary camels, emerging infectious diseases, Ixodidae, *Hyalomma* ticks, vector-borne infections, tick-borne disease, Sahara, zoonoses, parasites, viruses

## Abstract

Serosurvey results for Crimean-Congo hemorrhagic fever virus antibodies in dromedary camels in Algeria indicate that the pathogen is circulating endemically in desertic areas, despite the hostile environment. Thus, dromedaries are suitable sentinels for detecting human risk for Crimean-Congo hemorrhagic fever in desertic areas.

Crimean Congo hemorrhagic fever virus (CCHFV) is a tickborne *Orthonairovirus* that causes a potentially fatal hemorrhagic systemic disease in humans, Crimean-Congo hemorrhagic fever (CCHF). The virus is sustained in the ecosystem through wild and domestic animals, which act as tick amplification hosts and are asymptomatic (*1*). CCHF has recently increased in Africa and is emerging in new regions (*2*). However, knowledge of CCHFV epidemiology in North Africa is limited. 

In Algeria, CCHFV has been detected in ticks (*3*), but no human cases have been reported, probably because of inadequate surveillance (*2*). In recent years, breeding of dromedary camels (*Camelus dromedarius*) has increased (*4*); dromedaries could be ideal indicators of CCHFV circulation because they are widely distributed across Algeria and are commonly reared in open environments with exposure to ticks. To evaluate the distribution of the virus and the potential risk factors associated with CCHFV exposure, we conducted a serosurvey of CCHFV in dromedaries from the northeastern Saharan region of Algeria.

During 2020–2021, we collected 294 serum samples from dromedaries, of which 215 were from 23 different herds, and 79 samples from an abattoir, all from a region that included 4 provinces (wilayas): Biskra, El Oued, Touggourt, and Ouaregla. We tested samples for CCHFV antibodies by using a commercial kit (ID Screen CCHF Double Antigen Multi-species ELISA; IDvet, https://www. id-vet.com). We obtained data on risk factors associated with the individual animals (e.g., age) and management (e.g., breeding system) and evaluated their effect on CCHFV seropositivity with a mixed-effect logistic regression model with herd as a random effect, using R software (The R Project for Statistical Computing, https://www.r-project.org). We also collected data with regard to patterns of movements (i.e., duration and seasonality) for grazing. To evaluate the characteristics of the environment in which the dromedaries were reared, we created a buffer area around each herd (with a 100-km radius, considering the pattern of movements), and obtained the features of the land cover (*5*).

Animal-level CCHFV seroprevalence was 75.5% (95% CI 69.9–79.8; 222/294), and herd-level seroprevalence was 95.7% (95% CI 93.3–98.0; 22/23) (Figure). Odds of being seropositive for CCHFV were higher among dromedaries bred in the traditional system (i.e., grazing outdoors all year) (7.2, 95% CI 1.1–48.7) and the semitraditional system (i.e., grazing outdoor all year except for winter) (4.5, 95% CI 1.04–19.1) than among animals kept in permanent confinement. Odds of being seropositive were also higher among animals 4–10 years of age (6.5, 95% CI 2.2–19.5) and animals >10 years of age (14.9, 95% CI 3.2–69.4) than among animals <4 years of age (Table). The environment in which the dromedaries grazed was composed essentially of sandy desert (48.0%), bare areas (26.1%), and consolidated bare areas (i.e., bare rocks or stones) (11.5%).

**Figure:**
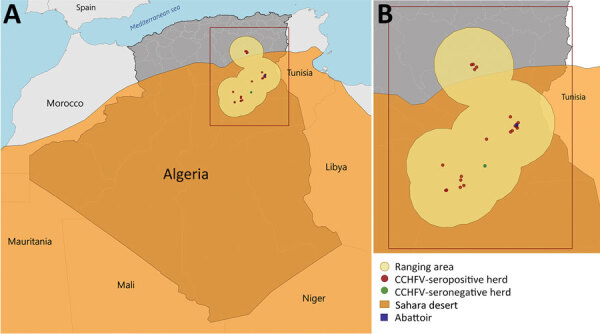
Locations of CCHFV seropositive and seronegative dromedary camel (*Camelus dromedarius*) herds, with ranging areas of 100 km. A) Ranging areas of herds in Algeria and coverage of the Sahara. B) Closer view of ranging areas, showing CCHFV-positive and -negative camel herds and location of abattoir. CCHFV, Crimean-Congo hemorrhagic fever virus.

**Table:** Results of mixed-effect logistic regression model used in study of Crimean-Congo hemorrhagic fever virus seropositivity among dromedary camels, Algeria, 2020–2021*

Fixed effects	Odds ratio (95% CI)	p value
Breeding system		
Intensive	Reference	NA
Traditional	7.24 (1.08–48.7)	0.042
Semitraditional	4.47 (1.04–19.1)	0.044
Age, y		
<4	Reference	NA
4–10	6.51 (2.17–19.5)	0.0008
>10	14.88 (3.19–69.4)	0.0006
AIC	178.5	NA
R^2^	0.50	NA

*The model included breeding system (3 categories) and age (3 categories) as the explanatory variables and herd as a random effect. AIC, Akaike information criterion; NA, not applicable.

Our results show that exposure of dromedaries to CCHFV is widespread; seroprevalence was high at both the herd and individual animal levels. Individual seroprevalence (74.8%) was similar to that reported in other countries of North Africa (*6**,**7*), suggesting that dromedaries may play a role in the epidemiology of CCHFV. Increased age was associated with higher CCHFV seroprevalence, which probably indicates that the virus has been endemic for some years. However, our finding that 22/56 (39.3%) dromedaries <1 year of age were seropositive indicates intense recent CCHFV circulation in the area. Traditional and semitraditional breeding systems increased the likelihood of CCHFV seropositivity because of the increased probability of exposure to CCHFV-infected ticks. Traditionally, dromedaries have been reared in constant movement across large pastoral areas in the Sahara desert, but nomadism is being replaced by transhumance (i.e., shorter and seasonal movements), especially in the northeastern Saharan region of Algeria (*8*). None of the herds in our study was nomadic; most movements were <4 days, implying that seropositive animals were exposed within the study area. Therefore, CCHFV circulation in dromedaries from this region most likely maintains itself without the need for repeated introductions from neighboring areas.

Attempts to map the distribution of CCHF risk have indicated that the areas at risk in Africa were basically restricted to the sub-Saharan region, where CCHF was associated with the presence of shrub or grassland (*9*). Because we found that bare or sandy desert areas are also favorable for CCHFV transmission, more studies are needed to evaluate animal hosts and tick vectors involved in CCHFV spread in those areas. Moreover, in the northeastern Saharan region of Algeria, the practice of breeding dromedaries in peri-urban areas has recently increased (*10*), which could increase the risk for human exposure to CCHFV. Developing a robust surveillance system for detecting human cases and monitoring CCHFV infection in peri-urban dromedaries is essential for early detection of the risk and implementation of preventive measures**.**
